# Phase I study of cisplatin, irinotecan, and epirubicin administered every 3 weeks in patients with advanced solid tumours

**DOI:** 10.1038/sj.bjc.6601147

**Published:** 2003-08-12

**Authors:** X Chen, A M Oza, Z Kusenda, Q-L Yi, D Kochman, M J Moore, A J Davis, L L Siu

**Affiliations:** 1 Department of Medical Oncology and Hematology, Princess Margaret Hospital, University Health Network, 610 University Avenue, Toronto, Canada M5G 2M9

**Keywords:** phase I study, irinotecan, cisplatin, epirubicin

## Abstract

This phase I study was conducted to determine the recommended phase II doses, safety profile, and antitumour activity of a combination regimen of cisplatin, irinotecan, and epirubicin administered every 3 weeks in patients with advanced solid tumours. Cisplatin and epirubicin were given at fixed doses of 50 and 60 mg m^−2^, respectively. The irinotecan dose was escalated at 10 mg m^−2^ increments from a starting dose level of 70 mg m^−2^. Epirubicin, irinotecan, and their metabolites were measured with HPLC methods. In all, 35 patients received 141 courses of treatment. Irinotecan dose was escalated in seven cohorts up to 130 mg m^−2^, and then finally de-escalated to 110 mg m^−2^. The dose-limiting toxicity was neutropenic fever. Nonhaematologic toxicities included mild to moderate nausea/vomiting, diarrhoea and fatigue. Of 34 patients with evaluable disease, one patient had a complete response and nine patients had partial response, yielding an overall response rate of 29.4%. Pharmacokinetic parameters of epirubicin were not affected by the sequence of drug administration. However, the AUCs of irinotecan and its metabolites were increased significantly when irinotecan and epirubicin were administered concurrently. This combination regimen has promising broad antitumour activity, and will be further evaluated in phase II studies in multiple tumour types.

DNA topoisomerases I and II are nuclear enzymes that regulate the torsional strain of supercoiled DNA double helix during critical cellular processes such as replication, transcription, recombination, and repair (Stewart *et al*, 1998; Li and Liu, 2001; Topcu, 2001). Several antineoplastic agents have been found to exert their cytotoxic effects by inhibiting topoisomerases. Irinotecan hydrochloride (CPT-11; CAMPTOSAR®), a semisynthetic camptothecin, is a potent inhibitor of topoisomerase I (Iyer and Ratain, 1998). It has demonstrated a broad spectrum of antitumour activity and has become a standard treatment of metastatic colorectal cancer recently (Saltz *et al*, 2000; Vanhoefer *et al*, 2001). Inhibitors of topoisomerase II include epipodophyllotoxins (e.g. etoposide) and anthracyclines (e.g. doxorubicin, epirubicin). Topoisomerase inhibitors bind to and stablise the ‘cleavable’ complex formed between each topoisomerase enzyme and DNA, with either single-strand breakage (topoisomerase I) or double-strand breakage (topoisomerase II), ultimately resulting in cell death when an advancing DNA replication fork collides into such a complex and forms fatal breaks (Li and Liu, 2001; Topcu, 2001).

Since both types of topoisomerases are essential in many cellular processes, it is attractive theoretically to combine topoisomerase I and II inhibitors (Vasey and Kaye, 1997). Preclinical studies have shown that crossresistance to both topoisomerase I and II inhibitors is rare (Tsuruo *et al*, 1988; Matsuo *et al*, 1990). However, laboratory studies have shown conflicting results when topoisomerase I and II inhibitors were combined. Sequential administration of topoisomerase I and II inhibitors generally produces consistent additive or synergistic effects (Bertrand *et al*, 1991; Kim *et al*, 1992), whereas with concurrent administration, both antagonistic and synergistic effects have been observed depending on the cell line or tumour model studied (D'Arpa *et al*, 1990; Kaufmann, 1991; Kano *et al*, 1992; Pei *et al*, 1997; Eder *et al*, 1998). These approaches have been evaluated in several phase I/II clinical studies. Results with sequential administration of topoisomerase I and II inhibitors were generally disappointing with increased toxicities and minimal activity. Interestingly, concurrent administration of topoisomerase I and II inhibitors have been found to be active in clinical trials of small-cell lung cancer (SCLC) (Masuda *et al*, 1998), non-small-cell lung cancer (NSCLC) (Masuda *et al*, 1994; Oshita *et al*, 1997), and non-Hodgkin's lymphoma (NHL) (Saotome *et al*, 2000; Kancherla *et al*, 2001). Etoposide is the topoisomerase II inhibitor administered in most of these combination studies, while two trials used doxorubicin (Saotome *et al*, 2000; Dunphy *et al*, 2001).

Cisplatin reacts at the N7 position of guanine and adenine residues to form platinum–DNA adducts, which would lead to cell death if they are not excised and repaired. Repair of these platinum–DNA adducts requires unscheduled DNA synthesis. Topoisomerase inhibitors may, therefore, interfere with this repair mechanism, resulting in synergistic antitumour activity (Fukuda *et al*, 1996; Saltz *et al*, 1998). A number of phase I/II studies of irinotecan and cisplatin combination have demonstrated promising activity in NSCLC (Mori *et al*, 1997; Kobayashi *et al*, 1998), SCLC (Kudoh *et al*, 1998; Noda *et al*, 2002), gastric (Boku *et al*, 1999), oesophageal (Ilson *et al*, 1999), colorectal (Sato *et al*, 2001), ovarian (Adachi *et al*, 1999), and cervical cancers (Sugiyama *et al*, 2000).

It is possible that combining a topoisomerase II inhibitor, such as epirubicin, with cisplatin and irinotecan would further interfere with repair of platinum–DNA adducts, resulting in synergistic activity. Epirubicin, an epimer of doxorubicin, was selected in this study because it is as effective as doxorubicin at equimolar doses in advanced breast cancer, but is better tolerated, particularly less cardiac toxic than doxorubicin (Launchbury and Habboubi, 1993; Ormrod *et al*, 1999), and is commonly used in treating gastric cancer as part of the ECF (epirubicin, cisplatin, and infusional 5-fluorouracil) regimen (Findlay *et al*, 1994; Webb *et al*, 1997). This potential synergy has not previously been explored in preclinical or clinical studies.

We hypothesised that a combination chemotherapy regimen consisting of irinotecan, cisplatin, and epirubicin (PIE regimen) would have synergistic activity against solid tumours. The current phase I trial was conducted with the primary objective to determine the recommended phase II doses and safety profile of such a combination regimen in patients with advanced solid tumours. The secondary objective was to assess for preliminary evidence of antitumour activity in this patient population, and to evaluate the effect of epirubicin on irinotecan pharmacokinetics when used in combination.

## METHODS

### Patient eligibility

Patients were required to have histologically or cytologically documented advanced solid tumours refractory to conventional therapy or for whom there are no standard therapies. Eligibility criteria also included the following: (1) age 18 years or older; (2) ECOG performance status 0–2; (3) left ventricular ejection fraction (LVEF) more than 50% by MUGA scan; (4) adequate organ function with an absolute neutrophil count (ANC)⩾1.5 × 10^9^ l^−1^, platelet count ⩾150 × 10^9^ l^−1^, total serum bilirubin⩽1.5 × upper limit of normal (ULN), AST/ALT⩽3 × ULN, and serum creatinine⩽150 *μ*mol l^−1^ or calculated creatinine clearance ⩾60 ml min^−1^; and (5) negative pregnancy test for female patients with child-bearing potential. Radiotherapy was permitted, but must have been delivered to ⩽25% of the bone marrow, and must have been completed at least 4 weeks prior to study entry. Full recovery from radiation-induced toxicities was required. In addition, patients were required to have received ⩽2 prior chemotherapy regimens for metastatic disease and must not have received cytotoxic therapy within 4 weeks of study entry. If prior chemotherapy contained cisplatin, patients could not have experienced >grade 1 residual peripheral neuropathy. For patients who received prior chemotherapy with anthracyclines, the cumulative dose had to be ⩽300 mg m^−2^ of doxorubicin or equivalent anthracycline dose.

The study protocol was reviewed and approved by the Institutional Review Board of the Princess Margaret Hospital, University Health Network. All patients gave written informed consent before study entry.

### Treatment plan

All three drugs were administered sequentially (epirubicin, followed by cisplatin, followed by irinotecan) on Day 1 every 3 weeks with the exception of Course 2 as described below. Epirubicin and irinotecan were supplied by Pharmacia Canada Inc., Mississauga, ON, Canada. Cisplatin (Faulding Canada Inc., Montreal, QC, Canada) and epirubicin were given at fixed doses in this study with epirubicin given at a dose of 60 mg m^−2^ as an intravenous bolus, and cisplatin infused over 60 min at a dose of 50 mg m^−2^. Irinotecan was given over 90 min immediately after epirubicin and cisplatin in escalating doses as per protocol. During Course 2 only, irinotecan was given on Day 1 while epirubicin and cisplatin were given on Day 3 in order to evaluate possible pharmacokinetic interactions between irinotecan and epirubicin. Atropine was administered if diarrhoea, abdominal cramping or other symptoms of early cholinergic syndrome occurred within 1 h of receiving irinotecan. Diarrhoea and/or abdominal cramping were treated aggressively with loperamide as recommended (Abigerges *et al*, 1994). Dexamethasone and a 5-HT_3_ antagonist were administered to all patients as standard premedications unless contraindicated.

The initial starting dose of irinotecan was 70 mg m^−2^, and was escalated by increments of 10 mg m^−2^ between dose levels. Successive cohorts of at least three patients were treated at each dose level. If any of the first three patients experienced dose-limiting toxicities (DLT) at a dose level, three additional patients were enrolled to that dose level. If no more than one of six patients experienced DLT, then the next cohort of patients was treated at the next higher dose level. If two or more patients at any dose level experienced DLT, then that dose level was considered to have exceeded the maximum tolerated dose (MTD), and the dose level immediately preceding that was designated as the MTD (equivalent to the recommended phase II dose). An additional three patients were then enrolled at the MTD to further assess safety and side effects. Intrapatient dose escalation was not allowed.

Toxicities were assessed using the National Cancer Institute (NCI) common toxicity criteria (CTC, version 2.0). DLT was defined as any first course, ⩾grade 3 nonhaematologic toxicity except alopecia or inadequately controlled nausea and vomiting, ⩾grade 3 diarrhoea despite aggressive loperamide treatment, grade 4 neutropenia with fever, grade 4 thrombocytopenia, or dose delay of >2 weeks due to drug-related toxicity.

Patients were required to have an ANC ⩾1.5 × 10^9^ l^−1^, platelet count ⩾100 × 10^9^ l^−1^, and full resolution of gastrointestinal toxicities before initiation of each treatment course. The irinotecan dose was reduced by one dose level (10 mg m^−2^) for all subsequent treatment courses if a patient experienced grade 4 neutropenia, grade 3 thrombocytopenia, or grade 3 nonhaematologic toxicity in the previous treatment cycle. If a patient experienced neutropenic fever, grade 4 thrombocytopenia, or any grade 4 nonhaematologic toxicity in the previous treatment cycle, the irinotecan dose was reduced by two dose levels (20 mg m^−2^). Patients who experienced significant toxicity despite dose reduction by two levels were discontinued from the study. Growth factor support was not allowed on this study.

### Specimen collection and pharmacokinetic analysis

Heparinised venous blood samples were drawn at 0, 45, 90 min from the beginning of irinotecan infusion, then at 15, and 30 min and 1, 2, 4, 6, 8, 24, and 48 h after the end of irinotecan infusion; and at 0, 5, 15, 30, 60 min from the beginning of epirubicin infusion, then at 2, 4, 8, 24, and 48 h after the end of epirubicin infusion. Samples were centrifuged immediately at room temperature, and plasma was separated and stored in aliquots at −20°C until analysis.

Irinotecan, SN-38 and APC concentrations were determined using a high-performance liquid chromatography assay. Plasma samples were mixed with an internal standard (camptothecin) in acidified acetonitrile to precipitate plasma proteins, then incubated for 15 min at 40°C to convert analytes to their respective lactone forms. After addition of triethylamine (TEA) buffer (pH 4.2), samples were centrifuged and supernatants were transferred to an amber vial for injection (60 *μ*l) onto of a Zorbax-SB-C8 column (MacMod Analytical Inc., Chadds Fords, PA, USA) with a mobile phase of acetonitrile : 0.025 M TEA buffer (pH 4.2) (25 : 75%). The fluorescence detector was operated at an excitation wavelength of 372 nm, irinotecan and APC were monitored at an emission wavelength of 425 nm, while SN-38 and the internal standard were monitored at 535 nm. SN-38 glucuronide (SN-38G) concentrations were estimated as increases in SN-38 concentrations after incubation of plasma samples with *β*-glucuronidase. The lower limits of quantitation for irinotecan, SN-38, and APC were 1.28, 0.48 and 0.96 ng ml^−1^, respectively.

For analysis of epirubicin and epirubicinol, plasma samples were mixed with an internal standard (daunorubicin) and 1 ml of 0.02 M sodium biphosphate buffer (pH 4.0). Samples were added to C8 solid-phase extraction tubes and extracts were dried on an evaporator. They were reconstituted in the mobile phase of acetonitrile : 0.02 M sodium phosphate buffer (pH 2.5) (33 : 67%), and injected onto a C18 column (Phenomenex, Torrance, CA, USA). The fluorescence detector was set at excitation 480 nm and emission 560 nm. The lower limit of quantitation for both epirubicin and epirubicinol was 5 ng ml^−1^.

Pharmacokinetic parameters were determined by noncompartmental methods using WinNonlin (Version 3.0, Pharsight Corp., Mountain View, CA, USA). Since irintotecan pharmacokinetics is known to be linear for doses up to 180 mg m^−2^ (Mathijssen *et al*, 2001), area under the concentration *vs* time curve (AUC) was dose standardised. Pharmacokinetic parameters were compared using the paired *t*-test. A probability of *P*<0.05 was considered significant. The program S-PLUS for Windows (Version 6) (Insightful Corp., Seattle, WA, USA) was used for statistical analysis.

### Evaluations at baseline and during treatment

At study entry, baseline evaluations included complete medical history and physical examination, chest X-ray, ECG, MUGA scan, and radiological imaging of assessable disease. Routine laboratory evaluations including complete blood counts (CBCs) with differential and biochemical profiles were performed at the start of every treatment cycle. CBC with differential was repeated weekly throughout the study.

Radiological imaging was repeated every 6 weeks to assess tumour response until disease progression, completion of study treatment, or discharge of patient from the study. Tumour responses were assessed according to standard WHO criteria.

MUGA scan was repeated prior to each course once the cumulative dose of epirubicin exceeded 360 mg m^−2^. In patients who achieved an objective response or stable disease status without intolerable toxicity, up to eight courses of treatment could be given.

## RESULTS

### Patient characteristics

In all, 35 patients received treatment on this study from October 1999 to 2001. Patient characteristics are summarised in Table 1

**Table 1:** Patient characteristics

**Characteristic**	**Patients no.**	**(*n*=35) %**
*Age (years)*		
Median	53	
Range	34–69	
		
*Sex*		
Male	18	51.4
Female	17	48.6
		
*Performance* s*tatus, ECOG*		
0	14	40.0
1	19	54.3
2	2	5.7
		
*Tumour type*		
Gastric	10	28.6
Ovary	5	14.3
Unknown primary	5	14.3
Cervix	3	8.6
Bilary tract, hepatocellular	3	8.6
GIST	2	5.7
Small bowel	2	5.7
Oesophageal	2	5.7
Others^a^	3	8.6
		
*Prior therapy*		
Chemotherapy	15	42.9
Radiation therapy	1	2.8
Both chemotherapy and radiation therapy	4	11.4
None	15	42.9

a One of each of pancreas, lung, and head and neck cancers.

. The median age was 53 years (range: 34–69 years). In total, 18 men and 17 women were enrolled in the study. Of the patients, 94% had performance status of ECOG 0 or 1. Enrolled patients had primary tumours originating from a large variety of sites, the majority of which were from the gastrointestinal (51.4%) or gynaecological tract (22.9%). A total of 15 patients had received prior chemotherapy (10 patients one prior chemotherapy regimen, four patients two prior chemotherapy regimens, and one patient two prior chemotherapy regimens for metastatic disease and one prior adjuvant chemotherapy regimen), one patient had received prior radiation therapy, and four had received both therapeutic modalities. Of these, 15 patients were chemotherapy naïve.

### Determination of recommended phase II doses

The first three patients were treated at the initial dose level of irinotecan at 70 mg m^−2^, and all three patients tolerated the treatment well without DLT. At the second dose level of irinotecan at 80 mg m^−2^, one of the initial three patients enrolled at this dose level developed neutropenic fever during Course 1. Three additional patients were enrolled to this dose level, and one developed grade 3 diarrhoea, uncomplicated grade 4 neutropenia, and grade 4 thrombocytopenia. On review, the two patients with significant haematologic toxicity were both found to have slightly elevated baseline total serum bilirubin levels of 1.2 times, and 1.5 times ULN, respectively. Both of these patients had mild obstruction of hepatobiliary tracts due to disease (one with cholangiocarcinoma, and the other one with cervical cancer with metastases to the pancreas), which accounted for hyperbilirubinaemia. Two additional patients with normal baseline total serum bilirubin levels tolerated the 80 mg m^−2^ dose level without any DLT. Hence, of six patients with normal baseline total serum bilirubin levels who were treated at the irinotecan dose of 80 mg m^−2^, none developed DLT. Therefore, further enrollment was restricted to patients with normal total serum bilirubin levels. No DLTs were observed until irinotecan dose was escalated to 130 mg m^−2^.

At an irinotecan dose level of 130 mg m^−2^, one of the first three patients developed neutropenic fever during course 1, and one of two additional patients treated at that expanded dose level also developed neutropenic fever during Course 1. Therefore, the irinotecan dose was subsequently de-escalated. Three more patients were enrolled and treated at an irinotecan dose level of 120 mg m^−2^, two of these additional patients developed neutropenic fever during Course 1. The irinotecan dose was further de-escalated to 110 mg m^−2^, and three more patients were treated at this expanded dose level. No DLT was seen, and irinotecan at 110 mg m^−2^ every 3 weeks in combination with epirubicin 60 mg m^−2^, and cisplatin 50 mg m^−2^ was selected as the recommended phase II doses for this triple combination.

The actual study dose-escalation scheme is shown in Table 2

**Table 2:** Dose-escalation schedule and number of patients

**Dose level (mg m^−2^)**	**No. of patients**	**No. of courses**	**No. of patients with DLT/ total no. of patients treated at that dose level**	**Irinotecan dose intensity (administered/intended) (%)**
70	3	16	0/3	88.3
80	8	23	2/8	83.3
90	4	13	0/4	88.2
100	3	7	0/3	96.1
110	3	18	0/3	88.1
120	3	18	0/3	86.4
130	5	24	2/5	78.9
120	3	11	2/3	71.1
110	3	11	0/3	83.5
Total	35	141		

. A total of 141 courses of treatment were given, and the median number of course administered per patient was four with a range of one to eight. Out of 141 treatment courses, there were 45 episodes of treatment delays alone, 10 episodes of dose reduction alone, 21 episodes of both treatment delay and dose reduction, and one episode of cancelled treatment. Irinotecan received-to-intended dose intensity ratios ranged from 71.1 to 96.1%. Over 80% of the intended irinotecan dose was administered at all dose levels except at the highest two doses levels. At the recommended phase II dose level, the irinotecan received-to-intended dose intensity ratio was 86.4%.

### Toxicities

Overall, toxicities observed in this study were those expected from a combination of these three cytotoxic agents. Selected toxicities are summarised in Tables 3

**Table 3:** Summary of haematologic toxicities (for first and all courses)

		**First course grade 3/4 toxicity**			**Overall grade 3/4 toxicity (patient/course)**		
**Dose level (mg m^−2^)**	**Total no. of patients/courses**	**Neutropenia**	**Thrombocytopenia**	**Anaemia**	**Neutropenia**	**Thrombocytopenia**	**Anaemia**
70	3/16	0	0	0	2/6	1/3	0
80	8/23	6	1	1	8/20	2/5	2/5
90	4/13	2	0	0	2/6	0	0
100	3/7	3	0	0	3/5	0	0
110	3/18	2	0	0	3/7	0	0
120	3/18	2	0	0	2/3	0	1/1
130	5/24	4	0	2	4/15	1/2	4/6
120^a^	3/11	3	1	0	3/9	1/3	1/1
110^a^>	3/11	1	0	0	2/2	0	1/1

a Irinotecan dose was reduced to this level due to DLTs seen at higher doses.

and 4

**Table 4:** Summary of non-haematologic toxicities (for first and all courses)

		**First course grade 3/4 toxicity**			**Overall grade 3/4 toxicity (patient/course)**		
**Dose level (mg m^−2^)**	**Total no. of patients/courses**	**Nausea/vomiting**	**Diarrhoea**	**Fatigue**	**Nausea/vomiting**	**Diarrhoea**	**Fatigue**
70	3/16	0	1	1	0	1/1	1/1
80	8/23	1	1	1	1/1	1/1	1/1
90	4/13	2	0	2	3/5	0	2/4
100	3/7	0	0	0	1/1	1/1	0
110	3/18	0	0	0	1/1	0	0
120	3/18	2	0	0	2/3	0	1/1
130	5/24	1	0	1	1/1	0	1/1
120^a^	3/11	0	0	0	0	0	0
110^a^	3/11	0	0	0	0	0	0

a Irinotecan dose was reduced to this level due to DLTs seen at higher doses.

. Grade 3/4 haematologic toxicities included neutropenia in 29 patients (51.8% of all treatment courses); anaemia in nine patients (9.9% of all treatment courses), six of whom required transfusion of packed red blood cells; and thrombocytopenia in five patients (9.2% of all treatment courses), one of whom required transfusion of platelets. Five patients had seven episodes of neutropenic fever throughout their entire duration of study therapy, and all neutropenic fever episodes resolved with parenteral antibiotics treatment. No grade 4 nonhaematologic toxicities were observed in this study. Grade 3 nonhaematologic toxicities included fatigue, nausea, vomiting, and diarrhoea. However, these events were infrequent, occurring in ⩽10% treatment courses. Of note, no patient experienced grade 4 diarrhoea and only two patients experienced grade 3 diarrhoea during Course 1. There were no cases of treatment-related death. No clinically significant changes in LVEF on MUGA scans or development of symptomatic congestive heart failure were seen in any patients on this study.

### Antitumour activity

Response was not the primary end point of this phase I study; however, promising antitumour activity was observed. In all, 34 patients were evaluable for tumour response. The one nonevaluable patient had gastric carcinoma with ascites and peritoneal carcinomatosis as the only sites of disease. Overall, 10 patients achieved a major objective response: one complete response (2.9%, 95% CI: 0–8.5%), and nine partial responses (26.5%, 95% CI: 11.7–41.2%). The duration of response in the one patient with complete response was 7.3 months, and the median duration of response among the nine patients with partial responses was 5.7 months. One patient with jejunal adenocarcinoma demonstrated a complete response and received eight cycles of treatment. The other nine responding patients included three patients with gastric or gastro-oesophageal junction adenocarcinoma, three with squamous cell carcinoma of the cervix, one with ovarian cancer, one with hepatocellular carcinoma, and one with cancer of unknown primary. Among the 10 patients who had a response, three patients had received prior chemotherapy and radiation therapy, and two patients each received prior chemotherapy or radiation therapy. In addition, the patient with gastric adenocarcinoma who was not assessable for response had improvement in his peritoneal carcinomatosis and resolution of ascites after two cycles of treatment. He was progression free for 4 months and after disease progression, was retreated with the study regimen, and again remained progression free for eight additional months. Furthermore, 13 patients had stable disease, and had a median of four courses of treatment.

### Pharmacokinetics

Complete pharmacokinetic data for both Course 1 and Course 2 were available in 29 patients for irinotecan, and in 12 patients for epirubicin. Pharmacokinetic parameters of irinotecan, epirubicin, and their metabolites are summarised in Tables 5

**Table 5:** Pharmacokinetic parameters of epirubicin

	**Sequence**		
**Parameter**	**Irinotecan+epirubicin**	**Irinotecan → epirubicin**	***P*-value**
AUC_t_ (ng h ml^−1^)^a^	783±356^b^	723±296	0.7
AUC_inf_ (ng h ml^−1^)^a^	1032±370	910±368	0.5
CL (l h^−1^)	110±42	125±45	0.4
*V*_d_ (l)	3477±1937	2563±1122	0.08
*t*_1/2_ (h)	23.1±10.8	16.0±7.8	0.07
Epi-ol AUC_t_ (ng h ml^−1^)^a^	921±634	788±482	0.5

AUC_t_=area under the curve to last measurable concentration; AUC_0–∝_=area under the curve extrapolated to infinity; CL=total body clearance; *V*_d_=apparent volume of distribution; *t*_1/2_=elimination half-life.

a Dose standardised to 100 mg epirubicin.

b Values are expressed as means ±s.d.

and 6

**Table 6:** Pharmacokinetic parameters of irinotecan

	**Sequence**		
**Parameter**	**Irinotecan+epirubicin**	**Irinotecan → epirubicin**	***P*-value**
AUC_t_ (ng h ml^−1^)^a^	3261±1563^b^	2870±916	0.03
AUC_inf_ (ng h ml^−1^)^a^	3404±1761	2958±1018	0.02
CL (l h^−1^)	35.8±14.7	37.1±10.6	0.4
*V*_d_ (l)	532±247	574±160	0.3
*t*_1/2_ (h)	10.6±3.3	10.9±2.1	0.5
SN-38 AUC_t_ (ng h ml^−1^)^a^	102±54	78±36	<0.001
SN-38G AUC_t_ (ng h ml^−1^)^a^	558±480	378±223	0.005
Total SN-38 AUC_t_ (ng h ml^−1^)^a^	662±516	467±244	0.004
APC AUC_t_ (ng h ml^−1^)^a^	1144±866	725±460	0.001
AUC SN-38/AUC irinotecan	0.032±0.01	0.027±0.01	0.002
AUC SN-38G/AUC SN-38	5.5±2.9	5.1±2.1	0.3
AUC APC/AUC irinotecan	0.35±0.24	0.25±0.12	0.003

AUC_t_=area under the curve to last measurable concentration; AUC_0–∝_=area under the curve extrapolated to infinity; CL=total body clearance; *V*_d_=apparent volume of distribution; *t*_1/2_=elimination half-life.

a Dose standardised to 100 mg irinotecan.

b Values are expressed as means ±s.d.

**Figure 1:**
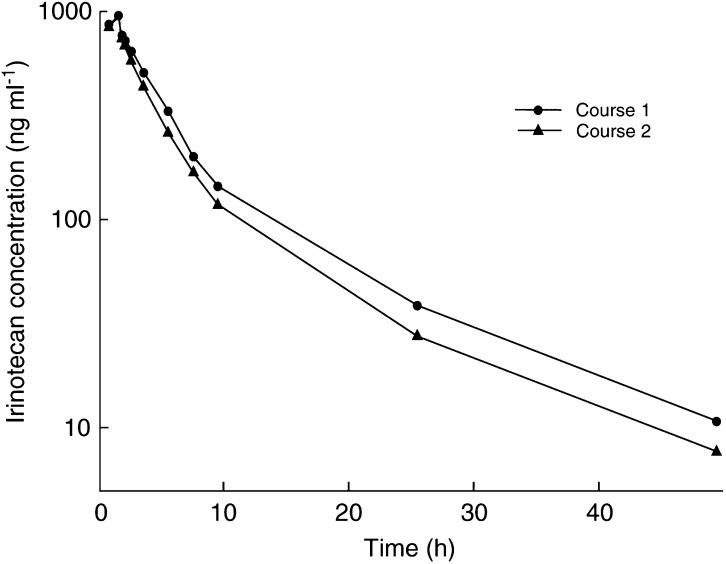
Dose standardised mean plasma irinotecan concentrations in Course 1 (administered immediately after epirubicin) and Course 2 (administered 2 days before epirubicin).

. Mean plasma irinotecan concentrations were plotted in Figure 1

.

Pharmacokinetic parameters of epirubicin were not affected by the sequence of drug administration (Table 5). However, when administered immediately after epirubicin, irinotecan AUC_t_ and AUC_inf_ increased significantly from 2870 and 2958 to 3261 and 3404 ng h ml^−1^, respectively. Similarly, AUCs for SN-38, SN-38G, total SN-38, and APC all increased significantly (Table 6). However, total body clearance, volume of distribution and *t*_1/2_ of irinotecan were not affected by coadministration with epirubicin.

At the irinotecan dose level of 80 mg m^−2^, one patient developed neutropenic fever, and one of the additional three patients enrolled at this dose level also developed prolonged neutropenia, grade 3 thrombocytopenia and grade 3 diarrhoea. These two patients were found to have elevated bilirubin levels at 1.2 and 1.5 times ULN. The ratio of epirubinol AUC to epirubicin AUC in Course 1 was 7 × that in Course 2 in the first patient. This patient developed neutropenic fever again on Course 2, although irinotecan dose was reduced, and bilirubin returned to normal. The second patient had the highest epirubicin, epirubinol, SN-38G, total SN-38, and APC AUCs in the study. Furthermore, compared to Course 2, SN-38G, total SN-38, and APC AUCs in Course 1 were >60% higher. This patient's bilirubin improved to normal, and she underwent four more courses of treatment with reduced irinotecan dose without any complication.

## DISCUSSION

The current phase I study was conducted to assess the feasibility of combining irinotecan, cisplatin, and epirubicin in an every-3-week schedule; the recommended phase II doses and side effects of this three-drug combination. This combination was conceived based on different mechanisms of antitumour activity of these agents, synergistic effects between irinotecan and cisplatin seen in preclinical studies, and broad efficacy of the irinotecan and cisplatin combination demonstrated in phase I/II clinical studies with multiple tumour types. Different and nonoverlapping toxicity profiles of these agents further support such a combination.

The two patients with elevated bilirubin levels treated at the irinotecan dose level of 80 mg m^−2^ provide an interesting insight into the potential role of biliary excretion in the elimination of epirubicin, irinotecan, and their metabolites. Irinotecan is converted to its active metabolite, SN-38, by the enzyme carboxylesterase. However, less than 10% of the administered irinotecan dose is converted to SN-38 (Slatter *et al*, 2000), which is further glucuronidated by the enzyme UGT1A1 to SN-38G and excreted via the biliary tract. Irinotecan-induced myelosuppression has been found to correlate with the plasma SN-38 exposure, as measured by the AUC. Over 60% of irinotecan and its metabolites are excreted via the biliary tract. Epirubicin is converted into several metabolites, including epirubicinol. Glucuronides of epirubicin and epirubincinol are subsequently excreted via the biliary tract. Impaired biliary excretion due to obstruction likely contributed to the DLT seen in these two patients. Enrollment of subsequent patients on study was restricted to patients with normal baseline total serum bilirubin levels, and no further DLT was observed until the irinotecan dose was escalated to 130 mg m^−2^. Therefore, it is possible that in patients with even mildly elevated bilirubin, biliary elimination of irinotecan, epirubicin and their glucuronide metabolites could be impaired, resulting in increased toxicity. Indeed, baseline plasma bilirubin and alkaline phosphatase levels have recently been found to correlate with irinotecan exposure and clearance (Raymond *et al*, 2002).

Pharmacokinetic interactions between irinotecan and other anticancer agents have been investigated in clinical studies of combination chemotherapy. No interaction was seen in majority of these studies (Saltz *et al*, 1996; Wasserman *et al*, 1999; Adjei *et al*, 2000; de Jonge *et al*, 2000b). The AUCs of both irinotecan and SN-38 were found to be lower when given together with carboplatin, suggesting increased clearance of irinotecan (Okamoto *et al*, 1998). In another study, the AUCs of both irinotecan and SN-38 were increased when given together with paclitaxel. However, the mechanism of this interaction was attributed to the paclitaxel vehicle, Cremophor EL (Yamamoto *et al*, 1999). In the present study, AUCs of irinotecan and its metabolites were significantly higher when administered immediately after epirubicin. In contrast, SN-38 AUC is not increased when irinotecan is coadministered with doxorubicin (Dunphy *et al*, 2001). Furthermore, ratios of SN-38 AUC to irinotecan AUC, and APC AUC to irinotecan AUC were also significantly higher, while that of SN-38G AUC to SN-38 AUC did not differ when irinotecan and epirubicin were administered on the same day. Total body clearance, volume of distribution, and *t*_1/2_ of irinotecan were not affected by epirubicin. Epirubicin pharmacokinetic parameters were not affected by the sequence of drug administration. One possible explanation is that epirubicin and its metabolites competitively inhibit biliary excretion of irinotecan and its metabolites. Therefore, irinotecan and epirubicin should only be administered concurrently after careful phase 1 studies to avoid potentially increased toxicities due to pharmacokinetic interaction between these two drugs.

The DLT in this study was neutropenic fever. Grade 3 or 4 neutropenia was the most common haematologic toxicity observed, with incidence ranging from 17 to 87% of treatment courses at various irinotecan dose levels. Even with the high incidence of neutropenia, the rate of neutropenic fever was low at 5% of treatment courses. Grade 3 or 4 thrombocytopenia and anaemia were seen in 9.2% and 9.9% of treatment courses, respectively.

No grade 4 diarrhoea was seen in this study, and grade 3 diarrhoea was rare at 2.1% of chemotherapy courses. This rate of grade 3 diarrhoea was consistent with rates reported in prior studies combining irinotecan and cisplatin (Saltz *et al*, 1998; de Jonge *et al*., 2000a). The lower frequency of grade 3/4 late diarrhoea in these three studies contrasts with the frequency of >20% grade 3/4 late diarrhoea observed in single-agent studies of CPT-11 administered on a weekly or every-3-week regimen and is presumably related to the lower CPT-11 doses when given in combination (Vanhoefer *et al*, 2001).

As expected, the combination of cisplatin, irinotecan, and epirubicin had antitumour activity in a variety of tumour types. One patient with jejunal adenocarcinoma had a complete response, and nine other patients had partial responses. With its broad spectrum of antitumour activity, this combination regimen would be worth further evaluation in multiple tumour types. Specifically, it will be further assessed in gastric or gastro-oesophageal junction carcinomas, in view of the demonstrated activity of the ECF regimen, as well as the irinotecan and cisplatin combination. The advantage of the PIE regimen is combining three of the most active agents against this malignancy while attempting to eliminate the inconvenience of infusional 5-FU.

Preclinical studies suggested synergistic effects on sequential administration of topoisomerase I and II inhibitors (Bertrand *et al*, 1991; Kim *et al*, 1992), but results from several phase II studies using this approach were disappointing with increased toxicities and no enhanced therapeutic efficacy (Ando *et al*, 1997; Herben *et al*, 1997). On the other hand, both synergistic and antagonistic effects have been reported when topoisomerase I and II inhibitors were administered concurrently in laboratory models (Pei *et al*, 1997; Eder *et al*, 1998). The results from our study are consistent with other studies of concurrent administration of topoisomerase I and II inhibitors, where encouraging antitumour activity has been reported in multiple tumour types (Masuda *et al*, 1994, 1998; Oshita *et al*, 1997; Saotome *et al*, 2000). The observed antitumour activity seen in this study might also be explained by the addition of cisplatin, and the use of epirubicin instead of etoposide as in most previous studies. Epirubicin, unlike etoposide, is not a pure topoisomerase II inhibitor, it also exerts its antitumour effects through other mechanisms such as free-radical generation and DNA intercalation.

In conclusion, the DLT in this phase I combination study of irinotecan, cisplatin, and epirubin (PIE regimen) administered every 3 weeks was neutropenic fever. The recommended doses for phase II studies are irinotecan 110 mg m^−2^, cisplatin 50 mg m^−2^, and epirubicin 60 mg m^−2^ on Day 1 with irinotecan administered immediately after epirubicin every 3 weeks in patients with good performance status and normal total serum bilirubin levels. The parabolic dose escalation encountered in this study suggests that the dose–effect relationship of this regimen is very steep, and the recommended phase II doses are unlikely generalisable for patients with poor performance status and abnormal hepatic function. Based on antitumour responses seen in this study, phase II studies of this combination in metastatic gastric cancer and cervical cancer have been initiated. In addition, further evaluation of optimal doses of this regimen in patients with mild liver dysfunction is ongoing to determine how best to modify doses in this special patient population.
